# Engineering species-like barriers to sexual reproduction

**DOI:** 10.1038/s41467-017-01007-3

**Published:** 2017-10-12

**Authors:** Maciej Maselko, Stephen C. Heinsch, Jeremy M. Chacón, William R. Harcombe, Michael J. Smanski

**Affiliations:** 1 Department of Biochemistry, Molecular Biology, and Biophysics and BioTechnology Institute, University of Minnesota – Twin Cities, Saint Paul, MN 55108 USA; 2 Department of Ecology, Evolution, and Behavior and BioTechnology Institute, University of Minnesota – Twin Cities, Saint Paul, MN 55108 USA

## Abstract

Controlling the exchange of genetic information between sexually reproducing populations has applications in agriculture, eradication of disease vectors, control of invasive species, and the safe study of emerging biotechnology applications. Here we introduce an approach to engineer a genetic barrier to sexual reproduction between otherwise compatible populations. Programmable transcription factors drive lethal gene expression in hybrid offspring following undesired mating events. As a proof of concept, we target the *ACT1* promoter of the model organism *Saccharomyces cerevisiae* using a dCas9-based transcriptional activator. Lethal overexpression of actin results from mating this engineered strain with a strain containing the wild-type *ACT1* promoter.

## Abstract (web summary)

Genetic isolation of a genetically modified organism represents a useful strategy for biocontainment. Here the authors use dCas9-VP64-driven gene expression to construct a ‘species-like’ barrier to reproduction between two otherwise compatible populations.

## Introduction

Engineering barriers to sexual reproduction between otherwise compatible organisms has numerous potential applications. Specific examples include preventing herbicide resistance genes from moving from cultivated to weedy plant varieties^1^, generating new mating incompatibilities to control pest populations, and for the safe study of gene-drives^2^. These applications could be achieved by engineering a speciation event, with speciation defined as reproductive isolating mechanisms that prevent genetic exchange between newly formed taxa^3, 4^.

Ideally, the introduction of species-like barriers would result in an engineered organism that behaves and can be propagated in an identical fashion to its non-modified counterpart. Changing the genetic code has been proposed as a means to accomplish this. Genetic recoding has been successful in *Escherichia coli* ^5, 6^ and *Saccharomyces cerevisiae* may soon follow^7, 8^. However, we are not likely to recode higher organisms with ease in the near future. Knocking down a copy of haploinsufficient genes has been used to generate hybrid depression^9, 10^, but not lethality. There are a handful of other examples of engineering genetic incompatibility in the literature. Chromosomal translocations that generate compound autosomes produce *Drosophila melanogaster* genetically incompatible with the wild-type, but these suffer from poor zygote viability^11^. A “synthetic species” of *D. melanogaster* ^12^ was developed by knocking out the glass transcription factor, and integrating a glass dependent killing module which is activated when mated with wild-type flies. Mating insects infected with different strains of *Wolbachia* bacteria can also result in embryonic lethality^13^. These approaches are either only applicable to a small number of species and/or dramatically change the engineered organism’s phenotype.

Several other forms of genetic incompatibility are in development or use for the control of pest species. These are mostly based on sterile insect technique which functions by releasing radiation or chemical sterilized males to find and non-productively mate with wild-type females^14^. For many insect species, *Wolbachia* infected males are incompatible with uninfected females; however, infected females are compatible with uninfected males so that sorting females from males must be highly efficient^13, 15^. Conditional lethal gene constructs exist which allow for the rearing of organisms under laboratory setting in the presence of a small molecule repressor. Offspring between released adult males and wild-type females are non-viable due to the absence of the small molecule. This approach has been successfully applied to the mosquito *Aedes aegypti* by the biotechnology company Oxitec^16^. A variation to this approach utilizes a conditional lethal gene that only kills female offspring^17^ which is in development for insect pests^18^ as well as invasive vertebrates species^19^.

These approaches will not doubt be valuable to controlling disease vector populations, however, unless the target species is driven to extinction, continuous release will be necessary to prevent reintroduction which may be prohibitively expensive in some settings. Population replacement strategies that result in disease resistant organisms occupying the vector’s niche^20, 21^ may be desirable in these cases. If the replacement population has been engineered to be disease resistant^22^, then it should ideally be incapable of reproducing with the wild-population to maintain the resistant genotype. *Wolbachia* infected mosquitoes are resistant to some arbovirus infections^23, 24^, but research is needed to determine if they may boost risk of other infections^25^. Combining engineered resistance with *Wolbachia* infection is an option, however, these strains would still be able to reproduce with the disease-vectoring population since *Wolbachia* infected females are usually capable of mating with uninfected males.

Multiple transgene biocontainment approaches have been explored beyond physical and/or temporal separation in plants including cleistogamy, maternal inheritance, gametic transgene excision, synthetic auxotrophy, and total sterility. However, each of these strategies has at least one major drawback that prevents wide-spread adoption. Cleistogamy, in which flowers never open and therefore must self-pollinate is not applicable to all species^26^. Cleistogamy may be possible to engineer in rice^27^ but it precludes the use of yield boosting hybrid seeds^28, 29^. Maternal inheritance of transgenes by plastid engineering is likewise not applicable to all species and pollen-mediated transfer of plastids at low frequencies may be common in many species^30^. The excision of a transgene from a pollen-expressed recombinase is highly efficienct^31^, however, control of recombinase activity interferes with normal propagation. Total sterility requires asexual propagation and is therefore not practical for many species. Any approach which uses additional chemical inputs to regulate lethal genes requires changes to normal cultivation techniques. Genome reprogramming to confer metabolic dependence on synthetic compounds^6^ is not yet possible in plants due to the large scale of genetic changes required. Furthermore, with the exceptions of cleistogamy and asexual propagation, these methods only prevent outward gene-flow. This is an important consideration since the unwanted flow of genes into genetically engineered plants can alter desirable traits.

Here we describe a broadly applicable approach to engineer species-like barriers to sexual reproduction. This method interrupts sexual reproduction between populations of different genotypes with minimal effects on growth and reproduction. Further, propagation of the engineered organisms does not require the use of exogenous inputs^32^. This technology may enable more scalable means for the containment of transgenic organisms, and provide additional tools to disrupt disease vector populations either through population reduction or replacement strategies.

**Fig. 1:**
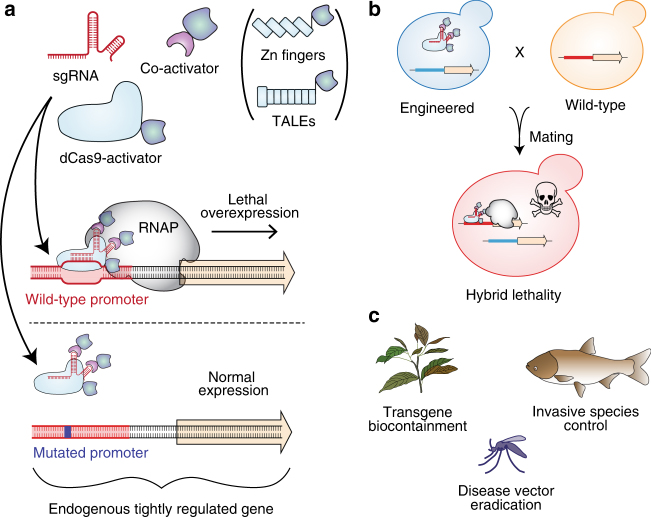
Overview of synthetic incompatibility. **a** Macromolecular components that constitute programmable transcription factors (above), and schematic illustration showing lethal gene expression from a wild-type but not a refactored promoter (below). **b** Illustration of hybrid lethality upon mating of wild-type (orange cell) and SI (green cell) parents. Macromolecular components are labeled in **a**, red DNA signifies WT promoter, and blue DNA signifies refactored promoter. Skull and crossbones indicates a non-viable genotype. **c** Possible applications for engineered speciation

In our approach, synthetic species-like genetic barriers are introduced via a relatively simple system (Fig. 1a–c) that utilizes programmable transcriptional activators (PTAs) capable of lethal overexpression of endogenous genes. Lethality in the engineered strain is prevented by refactoring the target locus, allowing the programmable activator to be expressed in the engineered strain. This activator serves as a sentinel for undesired mating events. Hybridization between the synthetically incompatible (SI) strain and an organism containing the transcriptional activator’s target sequence results in lethal gene expression (Fig. 1b). Because programmable transcription activators have been shown to work in many organisms^33–38^, this technology could be expected to readily transfer to higher organisms including plants^39^, insects^33^, and vertebrates^40^ (Fig. 1c).

We demonstrate a proof of concept for SI in the model yeast *Saccharomyces cerevisiae*. We introduce a mutation in the actin promoter and then engineer this yeast to express a PTA targeting the wild-type version of the actin promoter. Mating the engineered strain to the wild-type causes overexpression of actin and lysis of the hybrid cells.

## Results

### Targeting promoters for lethal overexpression

**Fig. 2:**
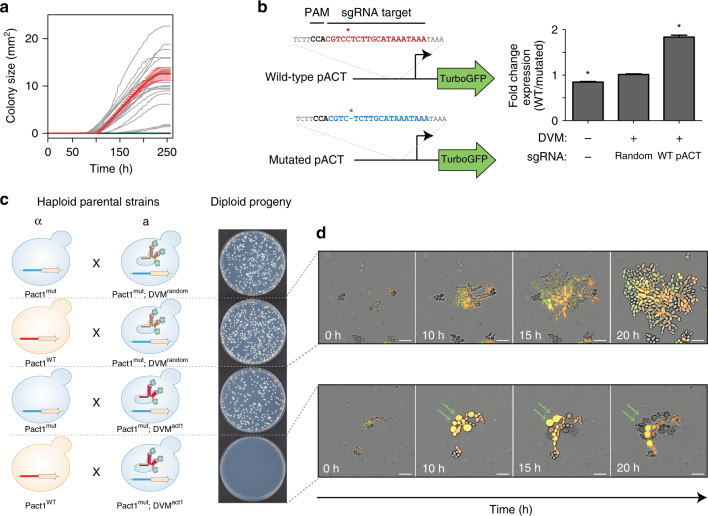
Engineering speciation by synthetic incompatibility. **a** Growth curves of yeast expressing DVM targeted to promoter regions of SI candidate genes. Random sgRNA control shown in red. Best *ACT1* targeting sgRNA in green. All others in gray. (*n* = 2 transformations, mean ± SEM, error bars omitted for gray lines for clarity). **b** (Left) Diagram of mutated and wild-type *ACT1* promoter-GFP constructs. (Right) GFP expression ratios with or without DVM and/or *ACT1* promoter specific sgRNA. (*p < 0.05, one-way ANOVA followed by Tukey’s post-test, *n* = 3 independent cultures, mean ± SEM). **c** (Left) Schematic representation of SI components present in haploid strain crosses and (right) the resulting diploid colonies. Results representative of three independent replicates. **d** Live-cell imaging time lapse of diploid cells from crossing RFP^+^ *MATα* with GFP^+^ *Mata* cells in a compatible (Top) and incompatible (Bottom) mating. Green arrows indicate cells which swell and lyse. The experiment was repeated twice. 20 µm scale bar

We demonstrate our approach in *S. cerevisiae* using a programmable transcriptional activation system composed of dCas9-VP64 combined with single-guide RNA (sgRNA) aptamer binding MS2-VP64 (referred hereafter as DVM) which is based on previously demonstrated strong activators^38, 41^. Appropriate target genes were identified empirically by using the DVM system to activate promoters of genes whose overexpression is reported to generate an ‘inviable’ phenotype in the *Saccharomyces* Genome Database^42^ (Supplementary Table 1). We designed sgRNAs to bind unique sequences immediately upstream of NGG protospacer adjacent motif (PAM) sites in a ~400 bp window upstream of predicted transcriptional start sites^43^ of candidate genes. Transformant growth rates were then measured for ~10.5 days. We identified several target sites which generated severely reduced growth rates and a single *ACT1* target which generated no growth (Fig. 2a, Supplementary Fig. 1). Additional screening for targets that generated no growth identified three more sites in the *ACT1* promoter and one combination of two guides targeting the *TUB2* promoter (Supplementary Fig. 1). For further analysis, we selected a target site on the bottom strand 300 nucleotides upstream of the *ACT1* transcriptional start site. The nine PAM distal nucleotides are predicted to be Forkhead transcription factor binding sites^44^.

### Engineering SI strain

To generate a SI strain, we used Cas9^45^ to introduce a mutation by non-homologous end joining in the *ACT1* promoter. The mutated promoter differs from wild-type by a single cytosine deletion 3 bp upstream of the PAM site. There is no observable growth phenotype resulting from the mutated *ACT1* promoter (Supplementary Fig. 2). We characterized transcription from the mutated promoter by expressing TurboGFP^46^ under the control of the wild-type or mutated *ACT1* promoters in the presence or absence of DVM (Fig. 2b and Supplementary Fig. 3). There was a slight increase in TurboGFP expression from the mutated promoter in the absence of DVM. However, no change was found with a non-targeting sgRNA. TurboGFP expression was 1.8-fold higher from the wild-type *ACT1* promoter than the mutated promoter when DVM was guided by a sgRNA targeting the wild-type promoter. Together, these results indicate that the mutation in the *ACT1* promoter does not substantially change native expression but prevents targeted transcriptional activation by DVM guided to the wild-type sequence. We completed construction of the SI strain by chromosomally integrating a DVM targeted to the wild-type *ACT1* promoter sequence in the strain containing the mutated *ACT1* promoter (i.e., Fig. 1b).

Next, we examined the genetic compatibility between the SI strain and a strain with the wild-type *ACT1* promoter. *S. cerevisiae* has haploid mating types *MAT*a and *MAT*α, and can be propagated as a haploid of either mating type or as a diploid after mating. We mated haploid strains with different auxotrophic markers and selected for diploids to determine mating efficiency (Fig. 2c). Mating a *MAT*a strain with the SI genotype but a random sequence sgRNA to a *MAT*α strain also containing the mutated *ACT1* promoter resulted in numerous diploid colonies (Fig. 2c). This shows that expression of the DVM machinery or mutation of the *ACT1* promoter do not prevent sexual reproduction. This same *MAT*a strain was also successfully mated to a *MAT*α strain carrying the wild-type *ACT1* promoter (Fig. 2c), as the random sequence sgRNA does not induce lethal overexpression of *ACT1*. We were also able to cross the *MAT*a strain with a complete SI genotype to a *MAT*α strain with the mutated *ACT1* promoter and obtain viable diploids (Fig. 2c). However, when the SI *MAT*a strain was mated with a *MAT*α strain containing the wild-type *ACT1* promoter, diploid colonies were seen only in low frequencies (Fig. 2c). This failed mating reflects the genetic incompatibility of the SI genotype with wild-type.

In order to understand how the concentrations of actin protein compared between conditions, we performed mating experiments and measured phalloidin stained F-actin content of diploid cells with flow cytometry. Diploid yeast resulting from non-permissive mating have 9.3-fold more F-actin than is present in diploids from permissive mating (Supplementary Fig. 4). This discrepancy from the modest 1.8-fold increase of promoter activity seen above suggests that the rate of actin degradation may not be increased to match the increase in synthesis.

We further investigated the genetic incompatibility using live-cell imaging (Fig. 2d, Supplementary Movie 1). Diploid yeast resulting from a permissive mating (Fig. 2c) are able to proliferate and produce a microcolony after 20 h (Fig. 2d, top). Diploids arising from the non-permissive mating of wild-type *ACT1* promoter yeast with the SI strain undergo a limited number of divisions before swelling and eventually lysing (Fig. 2d, bottom). These results are consistent with what we expect from uncontrolled cytoskeletal growth. However, the ability for these yeast to divide a few times before lysis may also provide opportunities for recombination and escape.

### Analysis of escape mechanisms

We next turned our attention to the colonies occasionally found when mating the SI strain to the wild-type. These colonies appeared at a frequency of 4.83 × 10^−3^ compared to mating with a compatible strain. Sanger sequencing the *ACT1* promotor found that most (3/5) originated from a cell homozygous for the mutated version of the promoter, suggesting that recombination had taken place between homologous chromosomes. Richardson et al.^47^ reported single-strand oligonucleotide homology directed repair frequencies of 7 × 10^−3^ in the presence of dCas9 in mammalian cell culture. Therefore, a similar mechanism may be responsible for the apparent mitotic gene conversion observed here. A fourth colony had a mutated MS2-VP64 activator and we were unable to locate mutations in the remaining colony.

Yeast is a useful system for demonstrating the molecular proof of concept for SI, however, there are challenges that will need to be addressed for applications in higher organisms. Identifying target genes that can be lethally overexpressed using PTAs is crucial. Since developing animal embryos are sensitive to concentration gradients of morphogens for proper body pattern formation, they have been found to be lethal when ectopically expressed^48–51^ and are likely to be ideal targets for SI. Indeed, Lin et al.^33^ demonstrated that dCas9-VPR can robustly activate several *D. melanogaster* morphogenic genes in vitro as well as cause embryonic lethality when targeted to the promoter of wingless and driving ectopic expression. Likewise, targeting genes that affect plant morphogenesis or immune response also holds promise for SI^52–54^. Transitioning SI to higher organisms will likely require high-throughput in vitro assays to identify PTA targets that are suitable for strong activation of candidate genes followed by in vivo tests for organismal lethality.

**Fig. 3:**
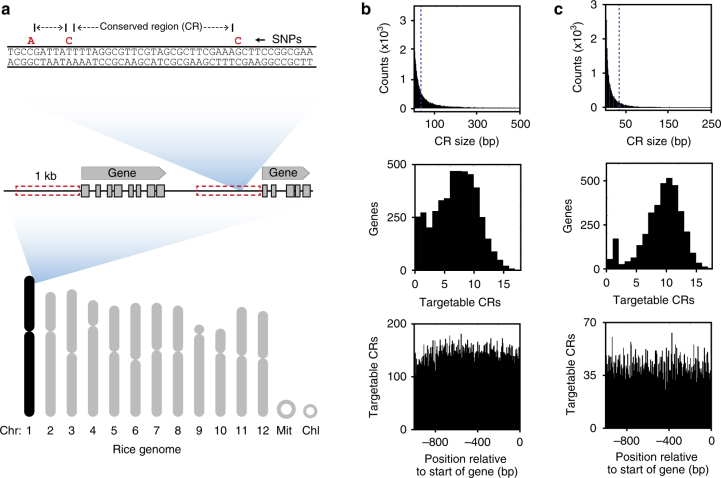
Promoter sequence diversity analysis in rice and fruit flies. **a** Schematic represenation of rice chromosome with illustration of SNP-free conserved region (CR) identification. Red box denotes 1 kb upstream of annotated genes from Chr1 in the *Oryza sativa* Nipponbare genome. **b** Number of conserved regions versus size is shown for all SNP-free conserved sequences in promoter regions of Chr1 (top, n = 65,500 CRs). Also shown are targetable CRs (defined as those larger than 30 bp) per promoter region (middle, *n* = 4487 genes), and the relative position of these targetable sequences relative to the start of the annotated gene (’0’) (bottom). **c** Equivalent data as in **b**, but for chromosome 2L from *Drosophila melanogaster*. (Total CRs in top panel = 215,705, total genes in middle panel = 3486). Spike at *x* = 1 for middle histograms is partially due to the counting of SNP-less promoters (entire 1 kb with no SNPs) as having a single CR

A possible pitfall to applying SI would be the presence of genetic polymorphisms at the target site that prevent PTA binding and therefore escape. Therefore, invasive species which underwent a recent genetic bottleneck^55, 56^ would pose less of a challenge than transgene containment between an engineered crop and a ubiquitous conspecific weed. We analyzed the promoter regions of rice (*Oryza sativa*) and *D. melanogaster*, both of which contain a substantial amount of variation^57, 58^. Our goal was to determine the frequency of single-nucleotide polymorphism (SNP)-free sequences in promoter regions which could potentially serve as dCas9-target sites using our speciation approach. An analysis was performed of promoter SNPs within chromosome 1 comparing the Nipponbare reference to all 45 US cultivars and land races available in the rice 3k SNP database^57^ (Fig. 3a, b). Despite substantial variation, there is an average of 6.5 SNP-free sequences per promoter region that are large enough to be targeted by a PTA (Fig. 3b). Likewise, an analysis of SNP data from 205 inbred wild strains of *D. melanogaster* collected from a food market in Raleigh, NC^59^ found an average of 8.8 SNP-free regions larger than 30 bp within 1 kb upstream of annotated genes from chromosome 2L (Fig. 3c). These targetable sequences are present with equal frequency throughout the 1 kb region upstream of annotated genes (Fig. 3b, c, bottom panel), allowing freedom to design diverse targeting sgRNAs.

Lastly, we have shown that a PTA can be linked to a positive selection module to ensure that it is expressed in the target organism. We replaced the promoter of a kanamycin selectable marker with a nucleotide sequence containing the PTA target site from pACT1 followed by a minimal promoter. Growth of yeast in the presence of kanamycin required expression of the same PTA used for engineering SI (Supplementary Fig. 5). Similar constructs linking the expression of an essential or selectable gene (e.g., herbicide resistance) may improve the robustness of SI.

## Discussion

In conclusion, we have presented the proof of concept for a, to the best of our knowledge, new approach to introducing defined genetic barriers to sexual reproduction. Synthetic incompatibility requires a single, phenotypically-neutral genomic edit, and the expression of a transcriptional activator targeting the unedited locus. Recently developed CRISPR-Cas9 based technologies should make it possible to apply synthetic incompatibility in a broad variety of sexually reproductive organisms. Recombination events between the target and mutant loci likely triggered by dCas9 binding indicate that applying this technology in higher organisms will require expressing dCas9 activators only in multicellular stages of life so that it is unlikely enough cells undergo recombination to rescue the whole organism. The period of transcriptional quiescence for the first cellular divisions in animal zygotes^60^ may therefore facilitate SI’s application.

Applying synthetic incompatibility to crops engineered to make biofuels or pharmaceuticals may allow for broader cultivation while preventing transgene flow to wild relatives or varieties used for human consumption. Synthetic incompatibility may also find applications in biocontrol of pest organisms by releasing SI males to reduce the fecundity of wild populations. As a form of lethal underdominance, synthetic species-like barriers hold promise as an approach to confine gene-drive systems and as a method to replace disease-vectoring populations of insects with non-vectoring insects^10, 61^.

## Methods

### Plasmids

Plasmid sequences can be found in GenBank with accession numbers listed in Supplementary Table 2 and primer sequences in Supplementary Data 1. Plasmid maps are found in Supplementary Fig. 6 and descriptions in Supplementary Table 2.

### Strains and media

Detailed information for all yeast strains can be found in Supplementary Table 3 and Supplementary Fig. 7. Yeast transformations were performed using the Lithium-acetate method^62^. Overnight liquid cultures were diluted to ~0.5 OD_600_ in 2X yeast extract-peptone-dextrose (YPD) medium and grown to ~2.0 OD_600_. The cells were then washed and mixed with DNA, lithium acetate, and salmon sperm carrier DNA and incubated at 42 °C for 40 min. The cells were then pelleted and plated onto dropout media or allowed 3–3.5 h of outgrowth in 2X YPD prior to plating onto G418 Sulfate selection. Chemically competent *E. coli* STBL3 (Thermo Fisher) was used for all plasmid cloning and propagation in LB media (MP) supplemented with appropriate antibiotics. Yeast were grown at 28–30 °C on plates or in liquid culture with 250 rpm agitation. Yeast were cultured in YPD (10 g/l yeast extract, 20 g/l peptone, 20 g/l dextrose), 2X YPD, or synthetic dropout (SD) media (1.7 g/l yeast nitrogenous base, 5 g/l ammonium sulfate, yeast SD media supplements (Sigma), 20 g/l dextrose). G418 sulfate resistant yeast were selected on YPD agar with 300–400 µg/ml G418 Sulfate. Counterselection for KlURA3 was performed using 1 g/l 5-Floroorotic acid.

### Screening candidate genes

Screening target genes was performed by transforming yeast strain YMM124 with pMM2-20-1 backbone vectors expressing sgRNA to candidate genes (Supplementary Tables 2 and 3). Transformations were plated onto SD-Ura in 6-well plates and incubated at 30 °C. To calculate growth rates of colonies on petri dishes^63, 64^, we scanned colonies as they grew using Epson Perfection V19 scanners in two hour intervals for 256 h. We used image analysis to track the areas of colonies as they grew. This entailed converting Red-Green-Blue (RGB) scans into Hue-Saturation-Value (HSV) colorspace, selecting the V channel, performing a background subtraction, smoothing, and using a threshold to identify biomass. The V channel was selected because it had the highest contrast with the background. The background was the first image in a time-lapse, before any colonies appeared. We smoothed images twice with a fine-grain Gaussian filter (sd = 1 pixel, filter width = 7 pixels) to remove noise. We used a single threshold for all images for consistency. Colony centers were identified by applying regional peak detection to a z-projection through time using the thresholded images. When colonies merged, we used these peaks to find the dividing line between colonies: the peaks were used as seeds in a watershed on a distance-transformed image. Once colony boundaries were identified, the number of “on” pixels within a boundary at each moment in time was counted as the colony’s area. We did not include in the analysis colonies which fell along the edge of the petri dish, which merged with colonies along the edge, or which had an ambiguous number of peaks within a large merged region. To calculate growth rates, we log-transformed the area-over-time data and fit a line in a 12 h moving window. The maximum slope in each time series was recorded as that colony’s growth rate. The growth rates were analyzed by one-way ANOVA followed by Tukey’s post-test to compare each condition to the random sgRNA control.

### Growth rate comparison

We inoculated 2 ml YPD with CEN.PK or YMM127 and placed them in a 30 °C shaker. After ~24 h, we inoculated 199 µl of YPD with 1 µl of culture in a clear, flat-bottom 96-well plate (Costar). Two technical replicates were included for each overnight culture. Plates were incubated at 30 °C in an Epoch 2 plate reader (BioTek) for 16 h with continuous shaking. The OD_600_ was measured every 10 min. Background subtracted OD measurements were plotted on a semilog graph and the slope was calculated from the linear portion of the graph (between 210 min and 330 min).

### Plate based mate assay

Haploid *MATa* yeast strain YMM134 and YMM155 were mated to *MATα* strains YMM125 and YMM141 by combining overnight cultures in YPD to an OD_600_ of 0.1 each in 1 ml YPD. The cultures were then incubated at 30 °C for 4 hours, washed once with water and 30 µl were plated onto SD-Ura/Leu dropout media.

### Flow cytometry

Flow cytometry for promoter analysis was performed using yeast strains YMM158 through YMM163. YMM158, YMM160, and YMM162 expressed TurboGFP driven by the wild-type *ACT1* promoter from plasmid pMM2-17-1. YMM159, YMM161, and YMM163 contained pMM2-17-2 and expressed TurboGPF from a mutated *ACT1* promoter. Overnight cultures grown in 2 ml SD-Complete media were diluted to an OD_600_ = 0.5 and grown for an additional four hours. Cells were collected by centrifugation, washed with DPBS, resuspended in DPBS, and placed on ice protected from light prior to analysis. Flow cytometry was performed using a LSRFortessa H0081 cytometer. At least 30,000 TurboGFP positive singlet events were collected per sample. The geometric means of GFP fluorescence intensity were compared using one-way ANOVA followed by Tukey’s post-test for pairwise comparisons.

F-actin content was analyzed by mating yeast strain YMM139 to YMM156 or YMM157 in SD-Trp using an OD_600_ = 1.0 of each strain. After 6.5 hr the media was replaced with SD-Ura/Leu/Trp for 24 h to select for diploids. Cells were subsequently fixed with 4% formaldehyde, permeabilized with 0.5% Triton X-100. F-actin was stained with phalloidin CF647 (Biotium) and then rinsed with PBS. Flow cytometry was performed using a BD FACS LSRII by collecting at least 10,000 events gated on singlet GFP and RFP positive cells and measuring the geometric mean of phalloidin fluorescent intensity then comparing groups by two tailed *t*-test.

### Live-cell imaging

Yeast strain YMM139 was mated separately with YMM156 or YMM157 in SD-Trp dropout media for 2 h, pelleted, and resuspended in SD-Ura/Leu/Trp. Mated yeast were loaded onto a CellASIC ONIX diploid yeast plate and supplemented with SD-Ura/Leu/Trp. Cells were imaged using a Nikon Ti-E Deconvolution Microscope System every 6 min for 20 h.

### Verification of yeast genomic mutations

Insertion of the MS2-VP64 cassette in the Lys2 locus was verified by PCR using primers MM_TA_CPCR_F and MM_Kan_CPCR_R which detect the presence of the transgene in the *Lys2* locus and MM_TA_CPCR_F and MM_TA_WT_CPCR_R which screen for the wild-type locus. (Supplementary Fig. 5a). Insertion of the sgRNA and dCas9-VP64 cassette into *Leu2* locus was verified by PCR using MM_DV_Leu2_CPCR_F and MM_DV_Leu2_CPCR_R which detect the presence of the transgene and MM_WT_Leu2_CPCR_F and MM_DV_Leu2_CPCR_R which detect the wild-type locus (Supplementary Fig. 5b). Mutations in the *Act1* promoter were detected by PCR amplifying a portion of the promoter using primers MM_Actg4_CPCR_F and MM_Actg4_CPCR_R. The gel purified amplicon was then Sanger sequenced using the MM_Actg4_CPCR_F primer.

### Promoter SNP analysis

All rice cultivars and landraces of US origin from the International Rice Center that were sequenced as part of the 3k SNP database^57^ (45 total) were analyzed for SNP distribution in promoter regions of Chromosome 1. One kb upstream of each annotated gene in the Nipponbare genome (GenBank) was extracted and searched for SNPs using the 3k SNP database data set. SNP-free regions between these loci, which represent sequences that are absolutely conserved across all genomes examined, were quantified to generate graphs in Fig. 3.

The fly data is from the Drosophila Genome Research Project^58^, and includes SNPs from 205 inbred wild strains collected from a food market in Raleigh, NC^59^. Promoter regions from chromosome 2L were used for the analysis of SNPs in a 1 kb region upstream of annotated genes, using the same methods described above for rice.

### Positive selection module

Yeast strains YMM130 and YMM131 were transformed with PCR amplicons of the positive selection modules and MS2-VP64 from plasmids pMM2-21-8 and pMM2-21-9 including flanking regions homologous to *lys2*. Cells were plated onto YPD supplemented with 300 µg/ml G418 to select for *lys2* integration events.

### Data availability

All data are available from the authors upon request.

## Electronic supplementary material

Supplementary Information

Description of Additional Supplementary Files

Supplementary Movie 1

Supplementary Data 1
