# Supplementary material for: Engineering species-like barriers to sexual reproduction
Source: Nat Commun. 2017 Oct 12;8:883. doi: 10.1038/s41467-017-01007-3 (PMC5638955; doi:10.1038/s41467-017-01007-3)
Supplement: Supplementary file 1 — Supplementary Information [file 41467_2017_1007_MOESM1_ESM.pdf]

Supplementary Figure 1

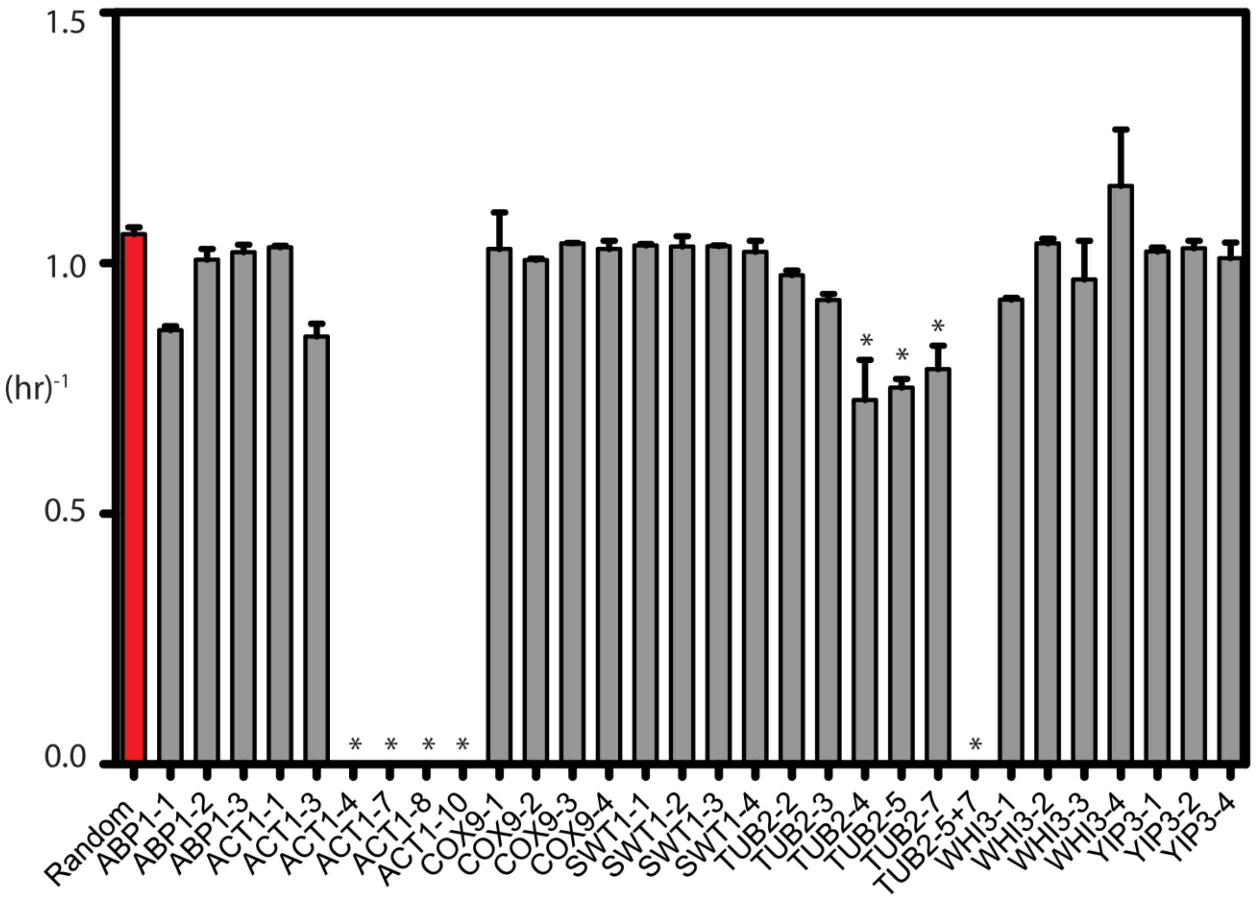

Supplementary Figure 1. | Growth rates of yeast expressing DVM targeted to promoter regions of SI candidate genes (n=2 independent transformations, mean +/- SEM). Red bar is the random sgRNA control. Asterisk indicates a statistically significant difference from the random sgRNA control (* indicates $p < .05$, one-way ANOVA and Tukey's post-test).

Supplementary Figure 2

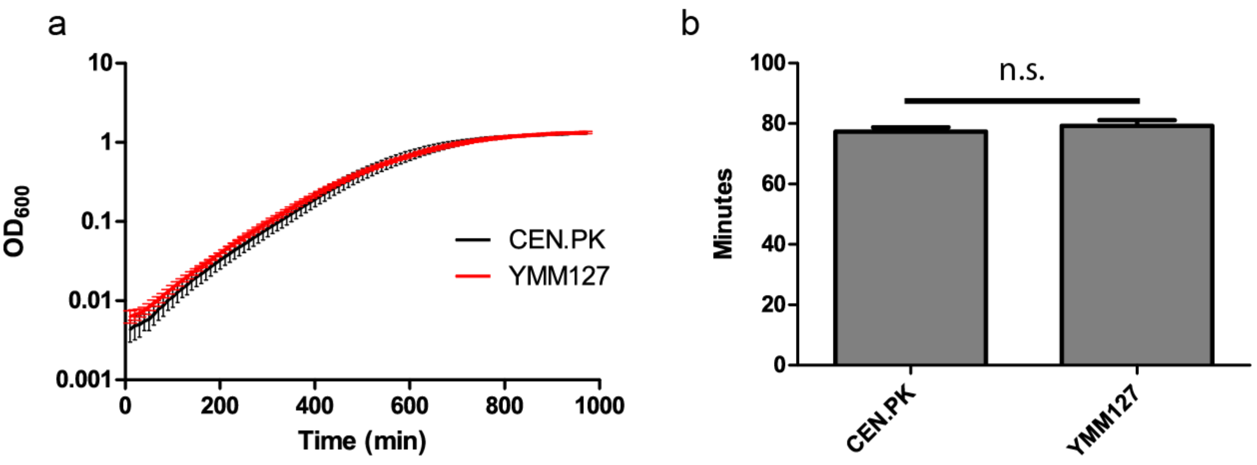

Supplementary Figure 2. Determining *ACT1* mutation's effect on growth rate | (a) Growth curves shown from strains with a wild-type (CEN.PK) and mutated (YMM127) actin promoters (n=3 independent cultures, mean +/- SEM). **(b)** Comparison of doubling time between CEN.PK and YMM127 ($p > .05$, two tailed t-test).

Supplementary Figure 3

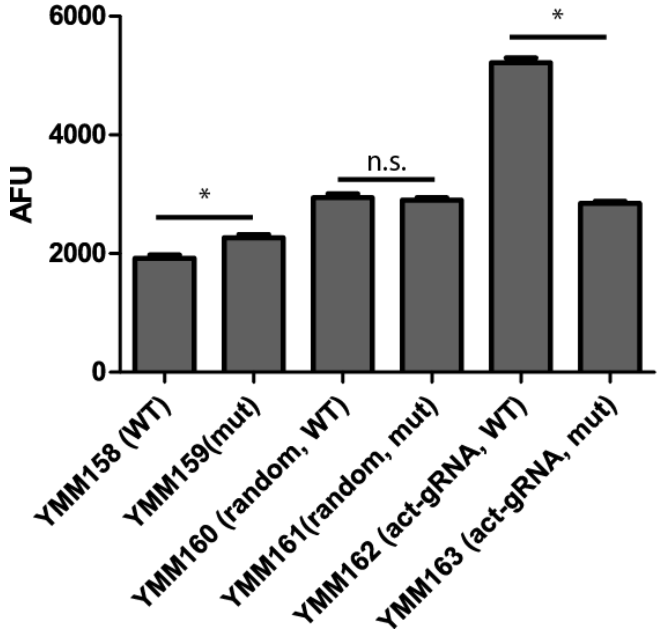

Supplementary Figure 3. Individual Cell Line Flow Cytometry Results | Average and SEM from three independent cultures. (* indicates $p < .05$, one-way ANOVA with Tukey's post-test)

Supplementary Figure 4

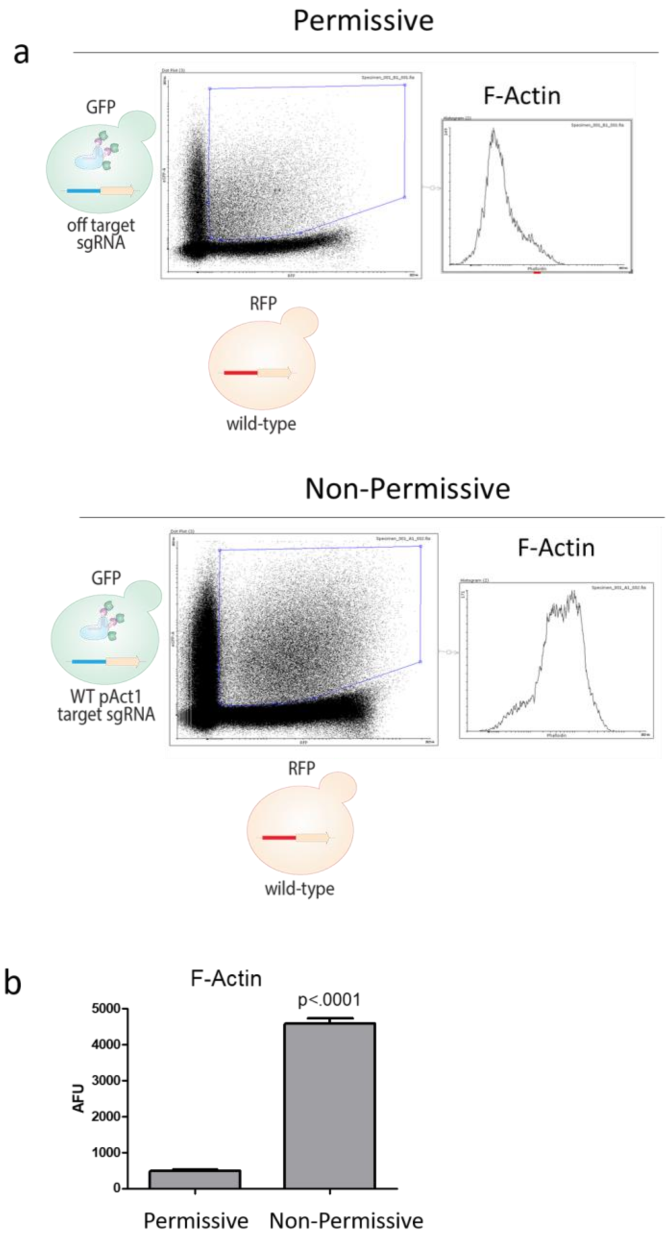

Supplementary Figure 4. Measuring F-Actin | (a) Representative dot plots and histograms from mating GFP and RFP expressing yeast in combinations permissive for diploid hybrids (top) and non-permissive (bottom). (b) Comparison of phalloidin stained F-actin in diploid hybrid cells. (n=3 independent matings, mean +/- SEM, two tailed t-test).

Supplementary Figure 5

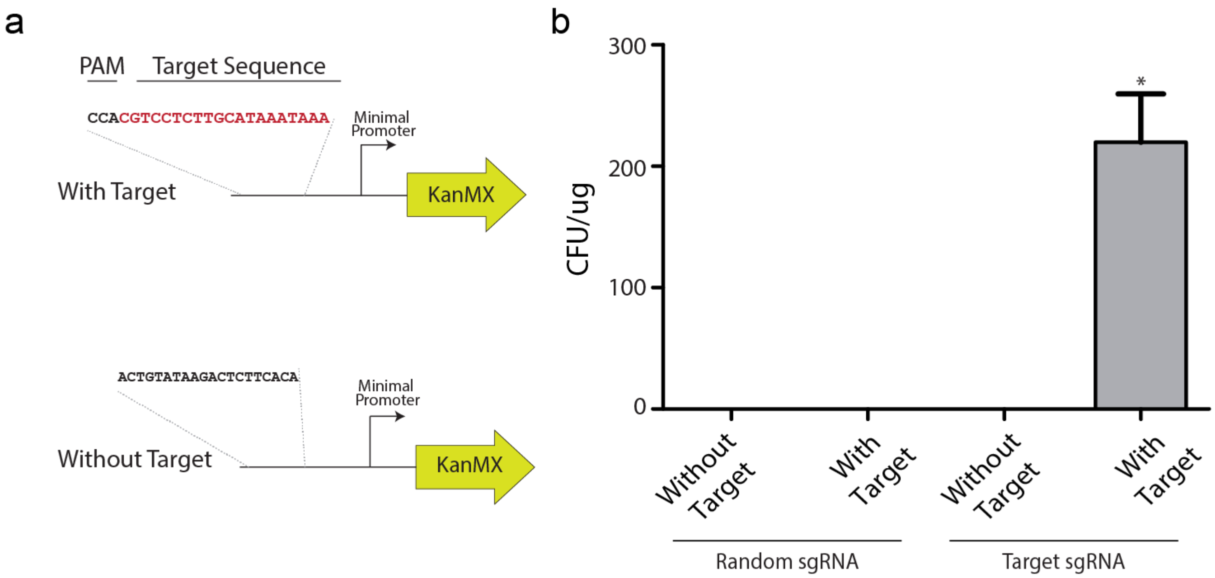

Supplementary Figure 5. Positive Selection Module | (a) Diagram of positive selection module containing the ACT1-4 target upstream of a minimal promoter and KanMX CDS. (b) Mean and SEM of colony forming units on G418 media from transforming yeast with integrating positive selection modules. Yeast expressed either DVM guided by a random sgRNA or from the ACT1-4 targeting sgRNA. (n=2 transformations. * indicates p < .05, one-way ANOVA with Tukey's post-test comparing far right column to all others).

Supplementary Figure 6

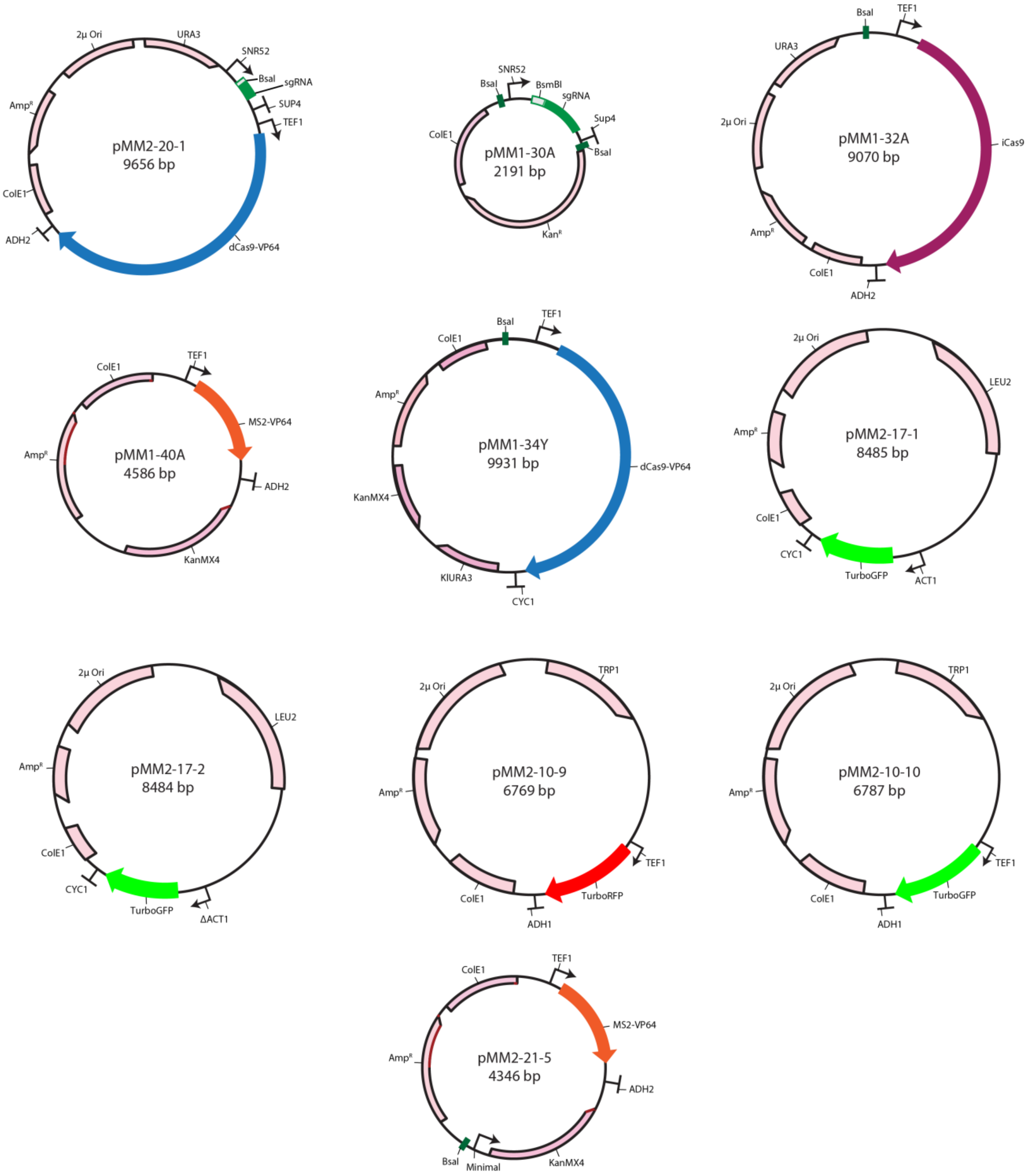

Supplementary Figure 6. Maps of key plasmids used in this study.

Supplementary Figure 7

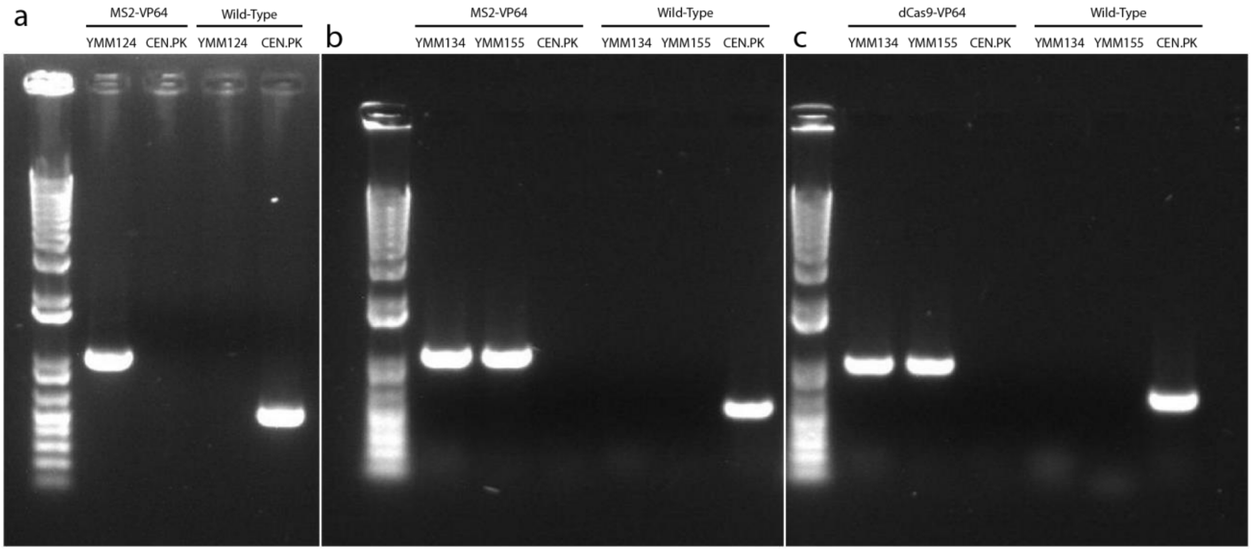

Supplementary Figure 7. PCR Verification of Genomic Modifications | (a) Results from PCR analysis of *Lys2* locus in YMM124 and CEN.PK wild-type control. (b) Results from PCR analysis of *Lys2* locus in YMM134, YMM155, and CEN.PK wild-type control. (c) Results from PCR analysis of *Leu2* locus in YMM134, YMM155, and CEN.PK wild-type control.

Supplementary Table 1

Supplementary Table 1: Target Genes

Gene	Function	Overexpression Phenotype [1]
ACT1	Actin. Cytoskeletal protein [2].	Inviable [3]
ABP1	Actin Binding Protein. Cytoskeletal regulation [4].	Inviable [3]
COX9	Subunit of cytochrome c oxidase [5].	Inviable [6]
SWT1	Endoribonuclease involved in mRNA quality control [7].	Inviable [8]
TUB2	β-Tubulin. Cytoskeletal protein [9].	Inviable [3]
WHI3	Regulator of cell cycle and cell size [10].	Inviable [10]
YIP3	Vesicular transport protein [11].	Inviable [12]

Supplementary Table 2

Supplementary Table 2: Plasmids used in this study

Plasmid	Description	Reference	GenBank
pMM2-20-1	Deleterious activation screening plasmid	this study	KX981587
pMM2-20-2 to 20-XX	pMM2-20-1 with spacers 1-XXX (Supplementary Table 4)	this study	
pMM1-30A	sgRNA 2.0 cassette with a BsmbI site for oligos. BsaI releasable.	this study	KX981578
pMM1-32A	iCas9 vector. Contains destination for sgRNA cassettes.	this study	KX981579
pMM1-40A	MS2-VP64 integration cassette template.	this study	KX981582
pMM1-34Y	Integrating dCas9-VP64 cassette. sgRNA cassette destination.	this study	KX981581
pMM2-4A	Template for integrating dCas9-VP64. ACT1 sgRNA.	this study	
pMM2-22-2	Template for integrating dCas9-VP64. Random sgRNA.	this study	
pMM2-17-1	WT pACT1 driven TurboGFP	this study	KX981585
pMM2-17-2	Mutated pACT1 driven TurboGFP	this study	KX981586
pMM2-10-9	TurboRFP expression plasmid.	this study	KX981583
pMM2-10-10	TurboGFP expression plasmid	this study	KX981584
pMM2-21-5	Empty Positive selection Module Plasmid	this study	
pMM2-21-8	Positive selection module with ACT1-4 target	this study	
pMM2-21-9	Positive selection module with no target	this study	
pCM159	Minimal promoter source for positive selection	[13]	
pCRCT	iCas9 source vector. Addgene #60621	[14]	
pICSL80004	TurboRFP source vector. Addgene #50325	[15]	
pICSL80005	TurboGFP source vector. Addgene #50322	[15]	
pCORE-UK	KlURA3 and KanMX4 source vector. Addgene #72238	[16]	
M-SPn-VP64	dCas9-VP64 source[a]. Addgene #48674	[17]	
pESC-Leu	Yeast replicative vector backbone source.	Agilent	
MS2-P65-HSF1_GFP	MS2 source plasmid. Addgene #61423	[18]	

[a] BsaI sites removed in this study.

Supplementary Table 3

Supplementary Table 3: Yeast strains used in this study

Name	Genotype[a]	Description
YMM124	*MAT*a, *lys2ΔMS2-VP64 KanMX4*	Used for screening growth defects caused by DVM
YMM125	*MATα LEU2*	Wild-type *ACT1* promoter strain used for mating experiments
YMM130	*MATa ACT1-Δ1 leu2ΔdCas9-VP64 Random sgRNA*	Random sgRNA strain used for positive selection module test.
YMM131	*MATa ACT1-Δ1 leu2ΔdCas9-VP64 ACT1 sgRNA*	ACT1-4 sgRNA strain used for positive selection module test.
YMM134	*MATa ACT1-Δ1 lys2ΔMS2-VP64 KanMX4 leu2ΔdCas9-VP64 Random sgRNA KlURA3*	Mutated *ACT1* promoter strain carrying DVM guided by random sgRNA
YMM139	*MATα LEU2* pMM2-10-9 (*TurboRFP TRP1*)	TurboRFP wild-type *ACT1* promoter strain.
YMM139b	*MATα LEU2* pMM2-10-9 (*TurboRFP TRP1*)	TurboRFP wild-type *ACT1* promoter strain.
YMM141	*MATα ACT1-Δ1 LEU2*	Mutated *ACT1* promoter strain used for mating experiments
YMM155	*MATa ACT1-Δ1 lys2ΔMS2-VP64 KanMX4 leu2ΔdCas9-VP64 ACT1 sgRNA KlURA3*	Synthetic incompatible strain
YMM156	*MATa ACT1-Δ1 lys2ΔMS2-VP64 KanMX4 leu2ΔdCas9-VP64 ACT1 sgRNA KlURA3* pMM2-10-10 (*TurboGFP TRP1*)	TurboGFP synthetic incompatible strain
YMM156b	*MATa ACT1-Δ1 lys2ΔMS2-VP64 KanMX4 leu2ΔdCas9-VP64 ACT1 sgRNA KlURA3* pMM2-10-10 (*TurboGFP TRP1*)	TurboGFP synthetic incompatible strain
YMM157	*MATa ACT1-Δ1 lys2ΔMS2-VP64 KanMX4 leu2ΔdCas9-VP64 ACT1 sgRNA KlURA3* pMM2-10-10 (*TurboGFP TRP1*)	TurboGFP mutated *ACT1* promoter strain carrying DVM guided by random sgRNA
YMM157b	*MATa ACT1-Δ1 lys2ΔMS2-VP64 KanMX4 leu2ΔdCas9-VP64 ACT1 sgRNA KlURA3* pMM2-10-10 (*TurboGFP TRP1*)	TurboGFP mutated *ACT1* promoter strain carrying DVM guided by random sgRNA
YMM158	*MATa* pMM2-17-1 (*pACT1- TurboGFP LEU2)*	No DVM strain with wild-type *ACT1* promoter driving TurboGFP
YMM159	*MATa* pMM2-17-2 (*pACT1-Δ1-TurboGFP LEU2)*	No DVM strain with mutated *ACT1* promoter driving TurboGFP
YMM160	*MATa ACT1-Δ1 lys2ΔMS2-VP64 KanMX4 leu2ΔdCas9-VP64 Random sgRNA KlURA3* pMM2-17-1 (*pACT1- TurboGFP LEU2)*	Random guide DVM strain with wild-type *ACT1* promoter driving TurboGFP
YMM161	*MATa ACT1-Δ1 lys2ΔMS2-VP64 KanMX4 leu2ΔdCas9-VP64 Random sgRNA KlURA3* pMM2-17-2 (*pACT1-Δ1-TurboGFP LEU2)*	Random guide DVM strain with mutated *ACT1* promoter driving TurboGFP
YMM162	*MATa ACT1-Δ1 lys2ΔMS2-VP64 KanMX4 leu2ΔdCas9-VP64 ACT1 sgRNA KlURA3* pMM2-17-1 (*pACT1- TurboGFP LEU2)*	Synthetic incompatible strain with wild-type *ACT1* promoter driving TurboGFP
YMM163	*MATa ACT1-Δ1 lys2ΔMS2-VP64 KanMX4 leu2ΔdCas9-VP64 ACT1 sgRNA KlURA3* pMM2-17-2 (*pACT1-Δ1-TurboGFP LEU2)*	Synthetic incompatible strain with mutated *ACT1* promoter driving TurboGFP

[a] All strains are derived from the CEN.PK background [19]: *ura3-52 trp1-289 leu2-3_112 his3 Δ1 MAL2-8C SUC2* except for YMM139b, YMM156b, and YMM157b which are derived from YNN216 [20]: *ura3-52 lys2-801 ade2-101.*

Supplementary References

[1] J. M. Cherry *et al.*, “Saccharomyces Genome Database: the genomics resource of budding yeast.,” *Nucleic Acids Res.*, vol. 40, no. Database issue, pp. D700-5, Jan. 2012.
[2] D. Gallwitz and R. Seidel, “Molecular cloning of the actin gene from yeast Saccharomyces cerevisiae.,” *Nucleic Acids Res.*, vol. 8, no. 5, pp. 1043–59, Mar. 1980.
[3] H. Liu, J. Krizek, and A. Bretscher, “Construction of a GAL1-regulated yeast cDNA expression library and its application to the identification of genes whose overexpression causes lethality in yeast.,” *Genetics*, vol. 132, no. 3, 1992.
[4] D. G. Drubin, K. G. Miller, and D. Botstein, “Yeast actin-binding proteins: evidence for a role in morphogenesis.,” *J. Cell Biol.*, vol. 107, no. 6, 1988.
[5] R. M. Wright, L. K. Dircks, and R. O. Poyton, “Characterization of COX9, the nuclear gene encoding the yeast mitochondrial protein cytochrome c oxidase subunit VIIa. Subunit VIIa lacks a leader peptide and is an essential component of the holoenzyme.,” *J. Biol. Chem.*, vol. 261, no. 36, pp. 17183–17191, 1986.
[6] R. Sopko *et al.*, “Mapping Pathways and Phenotypes by Systematic Gene Overexpression,” *Mol. Cell*, vol. 21, no. 3, pp. 319–330, 2006.
[7] S. Röther, E. Clausing, A. Kieser, and K. Strässer, “Swt1, a Novel Yeast Protein, Functions in Transcription,” *J. Biol. Chem.*, vol. 281, no. 48, pp. 36518–36525, 2006.
[8] M. Skružný, C. Schneider, A. Rácz, J. Weng, D. Tollervey, and E. Hurt, “An Endoribonuclease Functionally Linked to Perinuclear mRNP Quality Control Associates with the Nuclear Pore Complexes,” *PLoS Biol.*, vol. 7, no. 1, p. e1000008, Jan. 2009.
[9] N. F. Neff, J. H. Thomas, P. Grisafi, and D. Botstein, “Isolation of the β-tubulin gene from yeast and demonstration of its essential function in vivo,” *Cell*, vol. 33, no. 1, pp. 211–219, 1983.
[10] R. S. Nash, T. Volpe, and B. Futcher, “Isolation and characterization of WHI3, a size-control gene of Saccharomyces cerevisiae.,” *Genetics*, vol. 157, no. 4, pp. 1469–80, Apr. 2001.
[11] S. Otte, W. J. Belden, M. Heidtman, J. Liu, O. N. Jensen, and C. Barlowe, “Erv41p and Erv46p: new components of COPII vesicles involved in transport between the ER and Golgi complex.,” *J. Cell Biol.*, vol. 152, no. 3, pp. 503–18, Feb. 2001.
[12] J. Geng, M. E. Shin, P. M. Gilbert, R. N. Collins, and C. G. Burd, “Saccharomyces cerevisiae Rab-GDI displacement factor ortholog Yip3p forms distinct complexes with the Ypt1 Rab GTPase and the reticulon Rtn1p.,” *Eukaryot. Cell*, vol. 4, no. 7, pp. 1166–74, Jul. 2005.
[13] E. Garí, L. Piedrafita, M. Aldea, and E. Herrero, “A set of vectors with a tetracycline-regulatable promoter system for modulated gene expression in Saccharomyces cervisiae,” *Yeast*, vol. 13, no. 9, pp. 837–848, 1997.
[14] Z. Bao *et al.*, “Homology-Integrated CRISPR − Cas (HI-CRISPR) System for One-Step Multigene Disruption in Saccharomyces cerevisiae,” *Am. Chem. Soc.*, 2014.
[15] C. Engler *et al.*, “A Golden Gate Modular Cloning Toolbox for Plants,” *ACS Synth. Biol.*, vol. 3, no. 11, pp. 839–843, Nov. 2014.
[16] F. Storici and M. A. Resnick, “Delitto perfetto targeted mutagenesis in yeast with oligonucleotides.,” *Genet. Eng. (N. Y).*, vol. 25, pp. 189–207, 2003.
[17] K. M. Esvelt, P. Mali, J. L. Braff, M. Moosburner, S. J. Yaung, and G. M. Church, “Orthogonal Cas9 proteins for RNA-guided gene regulation and editing.,” *Nat. Methods*, vol. 10, no. 11, pp. 1116–21, Nov. 2013.
[18] S. Konermann *et al.*, “Genome-scale transcriptional activation by an engineered CRISPR-

Cas9 complex," *Nature*, vol. 517, no. 7536, pp. 583–588, 2014.
[19] J. . van Dijken *et al.*, "An interlaboratory comparison of physiological and genetic properties of four Saccharomyces cerevisiae strains," *Enzyme Microb. Technol.*, vol. 26, no. 9, pp. 706–714, 2000.
[20] M. Johnston and R. W. Davis, "Sequences that regulate the divergent GAL1-GAL10 promoter in Saccharomyces cerevisiae.," *Mol. Cell. Biol.*, vol. 4, no. 8, pp. 1440–8, Aug. 1984.
