# Supplementary material for: Engineering species-like barriers to sexual reproduction
Source: Nat Commun. 2017 Oct 12;8:883. doi: 10.1038/s41467-017-01007-3 (PMC5638955; doi:10.1038/s41467-017-01007-3)
Supplement: Supplementary file 2 — Description of Additional Supplementary Files [file 41467_2017_1007_MOESM2_ESM.pdf]

Description of Additional Supplementary Files

File Name: Supplementary Movie 1
Description: Live cell imaging time lapse of diploid cells from crossing RFP+ MATα with GFP+ MATa cells in a compatible (Left) and incompatible (Right) mating.

File Name: Supplementary Data 1
Description: Live cell imaging time lapse of diploid cells from crossing RFP+ MATα with GFP+ MATa cells in a compatible (Left) and incompatible (Right) mating.
